# Case report: clinical improvements observed in first off-label metreleptin treatment of a patient with atypical anorexia nervosa

**DOI:** 10.1007/s00787-023-02315-4

**Published:** 2023-10-24

**Authors:** Johannes Hebebrand, Jochen Antel, Triinu Peters

**Affiliations:** 1 Department of Child and Adolescent Psychiatry, Psychotherapy and Psychosomatics, University Hospital Essen (AöR), University of Duisburg-Essen, Wickenburgstrasse 21, 45147 Essen, Germany; 2 Center for Translational Neuro- and Behavioral Sciences, University Hospital Essen, University of Duisburg-Essen, Essen, Germany

**Keywords:** Hypoleptinemia, Metreleptin, Atypical anorexia nervosa, Weight phobia, Depression

## Abstract

Off-label metreleptin treatment resulted in cognitive, emotional and behavioral improvements of patients with anorexia nervosa, who presented with hypoleptinemia. We now report a case study of a 16-year-old female patient with atypical anorexia nervosa who was treated off-label with metreleptin for 11 days. She had lost 21 kg over 6 months. Her body mass index at referral for inpatient treatment was 20 kg/m^2^, her serum leptin level was just within the normal range (2.4 ng/ml). Dosing resulted in prominent improvements of mood and weight phobia entailing a comparatively brief inpatient treatment. The observed improvements are similar to those observed in patients with AN, suggesting overlapping mechanisms with respect to clinical effects induced by elevations of absolute or relative hypoleptinemia. Randomized controlled trials are warranted for both eating disorders.

## Introduction

Hypothesis-driven off-label metreleptin treatment of five patients with anorexia nervosa (AN) revealed psychological benefits including improved sleep and mood and a reduction of inner tension and preoccupation with both food and weight [3, 9, 14–17, 23]. These effects set in within 2–5 days after initiation of dosing; the dosing periods ranged from 6 to 24 days. Whereas symptoms tended to rebound albeit mostly not to levels prior to initiation of dosing, patients experienced the treatment as helpful. Patients felt as if being on a vacation from AN or being able to again look over the rim of the plate. Two of the published cases reported an increased appetite/hunger during treatment [9, 23], which does not support the role of leptin as an anorexigenic hormone in the state of starvation. The observed improvements show a substantial overlap with the improved symptomatology of the participants of the Minnesota Starvation Experiment [22] upon restricted refeeding. Accordingly, we postulated [16, 23] that hypoleptinemia induced by loss of fat mass likely contributes to the clinical symptomatology of ‘semi-starvation neurosis’ delineated by Ancel Keys and coworkers [22].

Treatment guidelines agree that weight recovery is a central goal of treatment of patients with AN [6, 19, 20, 24, 25].

The update of World Federation of Societies of Biological Psychiatry (WFSBP) guidelines from 2023 on the pharmacological treatment of eating disorders bestowed a weak recommendation to metreleptin for the use in anorexia nervosa [21]. These guidelines are lacking guidance on the pharmacological treatment of atypical anorexia nervosa.

Hypoleptinemia is a key endocrine feature of untreated inpatients upon referral [10, 12, 18]. Weight gain in patients entails improved psychological functioning and increased leptin secretion, suggesting that leptin might be a key humoral mediator.

A ‘significantly low body weight’ represents the first criterion (A criterion) for the diagnosis of AN according to DSM-5 [2]. DSM-5 does not refer to a strict cutoff for definition of a significantly low body weight. However, a BMI < 18.5 kg/m^2^ (between BMI 17.0 and 18.5 kg/m^2^ “clinical history or other physiological information should support this judgement”) and < 5^th^ sex and age-matched centile in adults and children and adolescents, respectively, is provided as guidance. DSM-5 defines Atypical AN (AAN) within the diagnostic category Other Specified Feeding and Eating Disorder as “all of the criteria for AN are met, except that despite significant weight-loss, the individual's weight is within or above the normal range”.

Research into AAN has increased over the last few years reflecting the substantial number of patients with an AN-like phenotype, who do not develop underweight [7, 8, 27]. According to some investigators, the disorder is potentially almost as frequent as AN [7]. Patients with AAN may also present with depression, which is a frequent comorbid disorder of AN [27]. We have previously discussed that psychological symptoms of starvation may account for the similar psychopathology of both AN and AAN [13]. Further, hypoleptinemia may underlie psychological symptoms of starvation [15]. We are unaware of any study that has specifically assessed serum leptin levels in a larger sample of patients with AAN.

We have treated an adolescent with AAN [2] and a BMI of 20 kg/m^2^ presenting with a serum leptin level in the lowest normal range off-label with metreleptin hypothesizing that as in AN hypoleptinemia contributes to the symptomatology. The patient suffered substantially from her symptoms and required inpatient treatment after outpatient therapy had not been successful in improving her condition.

## Case report

The female patient aged 16 years at referral reported a weight loss of 21 kg (recalled premorbid body weight 80 kg, estimated BMI 27.12 kg/m^2^) during the preceding 6 months due to both dietary restriction and excessive physical activity (body weight and BMI at referral 59 kg and 20.0 kg/m^2^). She suffered from a preoccupation with food and body weight, inability to give up her calorie-restricted diet, self-induced vomiting 1–2 times a day, strong drive for activity, inner tension, reduced concentration, and depressed mood. Binge eating episodes had not occurred. She had received compliments from friends for her weight loss, which she initially relished. However, as her symptoms worsened, she was no longer able to value such comments in light of her feeling ill. Amenorrhea had set in early after initial weight loss. The patient had experienced selective mutism as a child and currently reported symptoms of social phobia.

During the initial 3 weeks of inpatient treatment, the patient frequently withdrew to her room. Her mood was depressed; she frequently cried and felt homesick. Her eating behavior was restrictive; despite a target weight of 65 kg she continued to maintain a body weight of between 59 and 61 kg. Inner tension was particularly discernible during meals. All lab values including blood cell counts, liver enzymes, and electrolytes were within the normal range except for a subnormal serum level of magnesium. The serum leptin level of 2.4 ng/ml was just within the normal range (above 2 ng/ml). However, upon adjustment for BMI, Tanner stage and sex [5] this leptin level was between the first and fifth centiles.

In light of lack of progress during inpatient treatment, the patient and her parents agreed to off-label treatment with metreleptin. They were extensively informed of our experience with this drug in patients with AN and all known side effects of metreleptin including the FDA initiated risk management program due to the occurrence of lymphoma in three patients with generalized lipodystrophy and provided written informed consent. The patient was also informed of the necessity to eat three main courses and three snacks throughout the dosing period to avoid the development of hypoglycemia, which represents a side effect of metreleptin treatment.

**Fig. 1:**
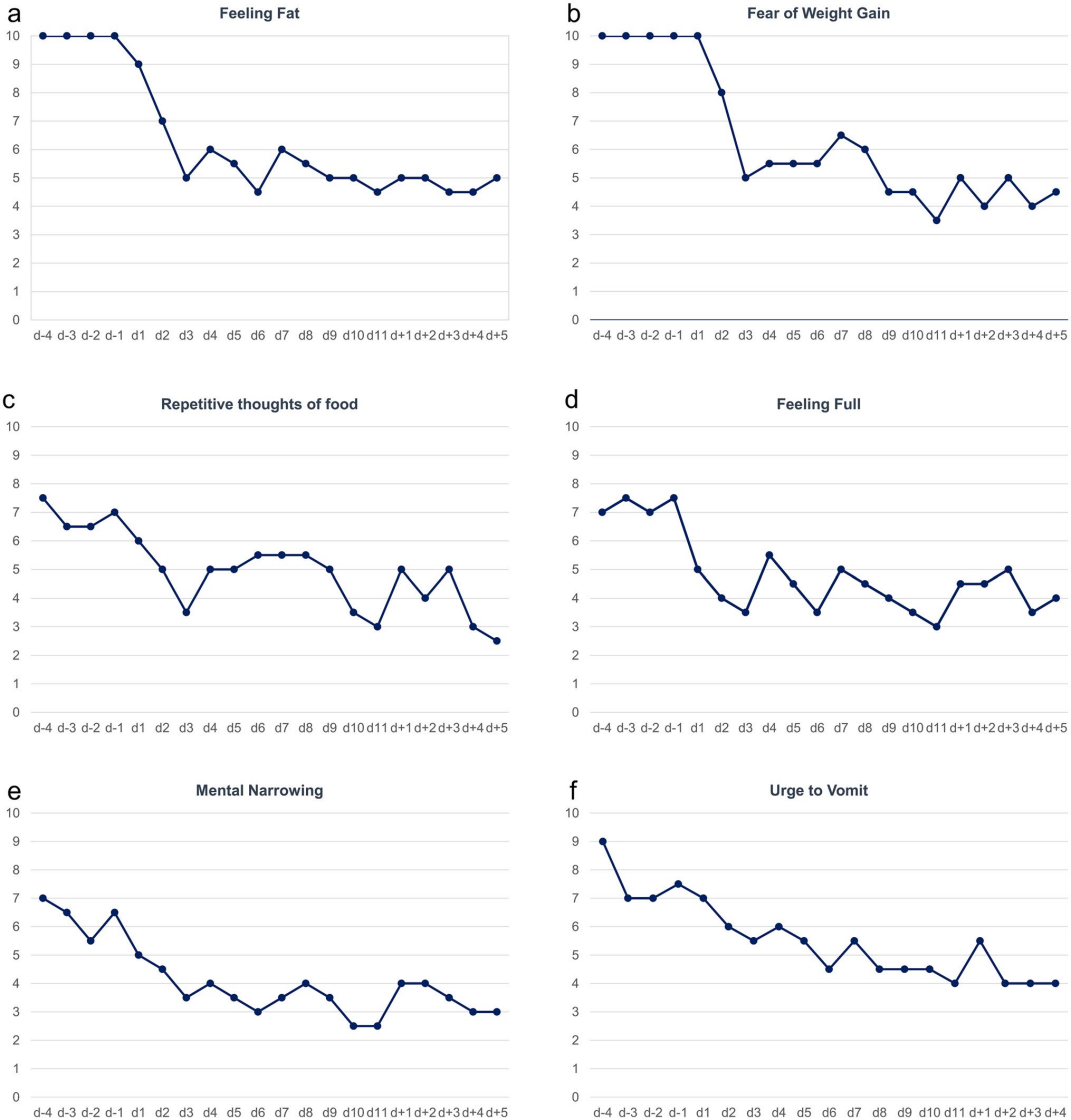
**a-f:** Visual analog scale results for six items. 10: maximal, 1: absent. Scales were filled in twice daily; dots represent respective daily means prior to dosing (-d4 to -d1), during dosing (d1 to d11) and after cessation of dosage (d + 1 to d + 5)

As in patients with AN, we used visual analogue scales (VAS) for the documentation of key emotions, cognitions and behaviors, which the patient filled in mornings and evenings (means are shown in Fig. 1). The scales ranged from 1 to 10 (1: symptom absent; 10: extreme). The patient additionally filled in the German versions of Beck Depression Inventory-II (BDI-II; [11] and Eating Disorder Inventory-2 (EDI-2; [26]). She was treated with 3.0 or 5.8 mg/daily, applied subcutaneously at 9:00 am. Otherwise, she received inpatient treatment as usual.

Follow-up and outcomes: Already during the afternoon of the first dosing day, another inpatient asked the index case why her mood had improved noticeably. Family members also reported an improved mood early during the dosing period. The patient remarked an undefined happiness. The BDI-II score dropped from 29 one day prior to dosing to 12 at dosing day 8. EDI-2 total scores dropped from the 95^th^ (d-1) to 85^th^ (d10) centile (Table 1).

**Table 1:** Beck Depression Inventory-II (BDI-II) and Eating Disorder Inventory-2 (EDI-2) scores one day prior to dosing (d-1), during dosing (d8, d10) and 10 days after cessation of dosing (d + 10)

	d-1	d8	d10	d + 10
**BDI-II**	29	12		17
**EDI-2**	335		290	272
Total raw score and centile; subscale centiles	95th centile		85th centile	80th centile
	Drive for Thinness 99		Drive for Thinness 85	Drive for Thinness 85
	Bulimia 75		Bulimia 60	Bulimia 50
	Body Dissatisfaction 95		Body Dissatisfaction 70	Body Dissatisfaction 80
	Ineffectiveness 95		Ineffectiveness 85	Ineffectiveness 80
	Perfectionism 90		Perfectionism 90	Perfectionism 80
	Interpersonal distrust 75		Interpersonal distrust 80	Interpersonal distrust 65
	Interoceptive awareness 90		Interoceptive Awareness 85	Interoceptive awareness 80
	Maturity fears 95		Maturity fears 85	Maturity fears 80

The VAS revealed reductions of ≥ 30% for fear of weight gain, feeling fat, feeling full, preoccupation with food, urge to vomit, and mental narrowing (Fig. 1 a-f) and drive for activity (data not shown). Depressed mood and inner tension were initially ranked at 6 and dropped to mostly 4; during the first days of the dosing period an improved mood was primarily reported during the afternoon and evening. At the end of treatment mood was also perceived as better during the morning. Suicidal thoughts (ranked as absent throughout the observation span), tiredness (average score throughout the observation period: 3.6), exhaustion (2.4), hunger (3.6), sleep quality (3.4), urge to binge (1.1), appetite (2.3), nausea (1.4), and interest in social interaction (6.5) remained largely unchanged (data not shown).

During and after the dosing period the patient did not vomit, which contrasted with its occurrence during initial inpatient treatment. She reported that respective urges only occurred once daily in contrast to 2–3 times prior to dosing. The strength of urges was reduced; she was better able to distract herself to overcome the urge. She stated perceiving her evening hunger as normalized in contrast to a reduced hunger prior to treatment (VAS for hunger in the evening averaged two prior to dosing and four after initiation of dosing). Preoccupation with food was estimated at 80% and 20–30% before and after the dosing period. In the evening, she no longer needed to reflect on what she had eaten during the day. Whereas she had had an extreme fear of weight gain, she was much better able to accept weight gain at the end of dosing. She reported thoughts of the importance of a sufficient amount of energy. Prior to dosing, she had also known of this importance, but had been unable to act upon it. At the end of the dosing period, she experienced weight gains as positive and weight loss as negative. Eating became easier during dosing. At the end of dosing, the patient stated that she slept very well; she perceived a feeling of tiredness in the evening as positive and as an indicator of a fulfilled day. Her ability to concentrate had improved. Whereas looking after her horse had been perceived as an obligation for many months, she again enjoyed this activity.

Both parents had observed an improved mood and a more normal eating behavior (e.g., patient voluntarily ate a slice of cake at home, which she had not been able to for a long time). They perceived their daughter as much more open during social exchanges, she would again chime in during discussions at home. The quantity of her speech had increased. At the horse stable, she for the first time spoke to people she did not like. The parents too reported that her care for her horse had increased and was perceived as fulfilling.

At the end of the dosing period the patient experienced stress due to the fact that the necessity of an outpatient treatment might not allow the family to undertake their vacation trip. She was nevertheless able to maintain course; intermittent downswings of her mood were not accompanied by deterioration of her eating behavior.

Body weight remained stable during the dosing period (60.9 kg one day prior to dosing; 60.4 kg last day of dosing; range for the initial 10 post-dosing days: 61.4–62.0 kg). She entered an intensive home treatment program; during the vacation in a foreign country, she relapsed and lost 3 kgs of body weight. She was able to regain weight after her return and weighed 63.2 kg upon the last observation (d + 60). Systolic and diastolic blood pressure was low throughout the pre-dosing and the dosing periods. Heartbeat rate dropped below 50 at eight of 20 (once daily) measurements prior to dosing day 2; from dosing day 2 to the end of the observation period two out of 15 measurements were below this value.

## Discussion

This case report is the first of a patient with AAN treated off-label with metreleptin. The results appear promising. Whereas we cannot rule out expectation effects, the clinical improvements appear similar to those observed in case studies of patients with AN. No adverse events were observed during or after the 11-day dosing period.

In contrast to previously treated patients with AN, the current case presented with a less severe clinical symptomatology. Nevertheless, inpatient treatment had to be initiated in light of failure of outpatient treatment and an inability of the patient to maintain daily functioning. Most of the observed improvements have previously been reported in patients with AN. We for the first time report a decrease in both vomiting and the respective urge. The current patient again reported an increased hunger during dosing. While tiredness was not a prominent symptom, the patient again reported sleeping very well [14].

Feeling fat and weight phobia were both ranked at the maximal value of ten in the VAS during the pre-dosing period. Upon completion of the dosing period, values were approximately halved. The values of approximately 5 at the end of the dosing period reflect the ongoing preoccupation with the former overweight, which appeared more realistic and not as a result of the entrapment in the eating disorder.

Our case report support to the hypothesis that symptoms of AAN can be starvation related, too. The adipokine leptin is a master hormone to trigger the neuroendocrine adaptation to starvation and may additionally act directly on leptin receptors enabling intracellular signaling throughout the brain and in peripheral tissues [1, 15, 16]. Obviously, the percentage of patients with AN or AAN who respond favorably to leptin receptor agonists remains to be determined. The fact that the current patient improved to an extent noticeable by the patient herself, parents and treatment team suggests that as in AN a large subgroup may respond to a clinically relevant degree. The leptin level prior to initiation of dosing was 2.4 ng/ml and thus minimally above the bottom value of the normal range (2 ng/ml). Upon adjustment of the leptin level for sex, pubertal status and BMI the concentration proved to be below the fifth centile [5]. It seems that the weight loss of approximately 20 kg in combination with the continuously restricted dietary intake accounted for this low leptin level. Studies are required to assess the range of leptin levels in patients with AAN. Based on a PubMed search using the key words “Atypical Anorexia Nervosa” (or “Eating Disorder Not Otherwise Specified”) and leptin, we were able to identify a single study which included two patients with atypical AN, whose leptin concentrations were ≤ 1.92 ng/ml [4]. The rapid response in our patient may indicate that patients with AAN respond even more quickly than patients with AN.

We argue that hypoleptinemia and the thus induced psychological and behavioral symptoms of starvation may represent a feature common to both AN and AAN. Accordingly, randomized controlled trials are warranted for both disorders to assess the efficacy and safety of metreleptin or other leptin analogues.

## Data Availability

Raw data are not publicly available to preserve individuals’ privacy.
